# Left ventricular flow kinetics and myocardial deformation following acute infarction: Additional predictive value of cardiac magnetic resonance four-dimensional flow for left ventricular remodeling post-ST-elevation myocardial infarction

**DOI:** 10.1016/j.jocmr.2025.101905

**Published:** 2025-05-07

**Authors:** Christel H. Kamani, May Lwin, Ioannis Botis, Mehak Asad, Noor Sharrack, Hadar Schapira, Arka Das, Peter P. Swoboda, Sven Plein, Rob J. Van der Geest, Erica Dall’Armellina

**Affiliations:** a Department of Biomedical Imaging Science, Leeds Institute of Cardiovascular and Metabolic Medicine, University of Leeds, Clarendon Way, Leeds LS2 9JT, UK; b Department of Cardiology, Lausanne University Teaching Hospital (CHUV), Rue du Bugnon 46, 1011 Lausanne, Switzerland; c Department of Radiology, Leiden University Medical Center, Albinusdreef 2, 2333 ZA Leiden, The Netherlands; d Department of Nuclear Medicine and Molecular Imaging , Lausanne University Teaching Hospital (CHUV), Rue du Bugnon 46, 1011 Lausanne, Switzerland

**Keywords:** CMR post MI, Global FT strain parameters, 4D flow parameters, Interaction of FT strain and 4D flow parameters, Prediction of LV adverse remodeling at 12 months post STEMI

## Abstract

**Background:**

The exact mechanism underlying myocardial maladaptive changes post ST-elevation myocardial infarction (STEMI) remains unclear. We sought to assess the impact of the tissue=flow interaction on the development of adverse cardiac remodeling 12 months(M) after acute STEMI.

**Materials and methods:**

Forty-nine first-STEMI patients (M:F = 26:13; mean age = 58 ± 10) prospectively underwent 3T cardiovascular magnetic resonance (CMR) acutely, at 3 months (3M) and 12M post-STEMI. The CMR protocol included left ventricular (LV) cine-images for LV end-diastolic (LVEDV) and end-systolic volumes, stroke volume (SV), and ejection fraction (LVEF); four-dimensional (4D)-flow and late gadolinium enhancement imaging. The 3M outcome measures included 4D-flow derived LV flow kinetic energy indexed to EDV (KE_iEDV_) and functional flow components [LV-KE_iEDV_, minimal- KE_iEDV_, diastolic- KE_iEDV_, and residual volume (RV), retained inflow, delayed ejection, direct flow (DF)]; global radial, circumferential, and longitudinal strain (GRS, GCS, GLS) by feature tracking (FT); infarct size (IS). Adverse LV remodeling (LV_remod_) was defined by a ≥20% increase in LVEDVi at 12M from baseline, in opposition to the non-remodeling group (LV_non-remod_). Association between SV, FT-strain, KE, and 4D flow parameters was assessed, as well as predictors of adverse remodeling at 12M post-STEMI.

**Results:**

There were 23 LV_remod_ patients. At 3M post-STEMI, LV_remod_ patients had significantly reduced LVEF, increased IS, abnormal FT-strain, systolic KE_iEDV_, DF, and RV compared to LV_non-remod_ patients. There was no significant difference in SV between the two groups. FT-strain parameters significantly correlated with DF (GRS: r = 0.62; GCS: r = −0.67; GLS: r = −0.58, all p < 0.001); RV (GRS: r = −0.56; GCS: r = 0.51; GLS: r = 0.53, all p < 0.001); peak-A-wave KE_iEDV_ (GRS: r = 0.38, p = 0.008; GCS: r = −0.30, p = 0.038; GLS: r = −0.29, p = 0.04); systolic KE_iEDV_ (GRS: r = 0.31, p = 0.033, GLS: r = −0.35, p = 0.012). DF outperformed conventional LV function parameters (SV and LVEF) in the LV_remod_ prediction. DF and IS were the only independent predictors of 12M adverse remodeling after adjustment for LVEF, SV, FT-strain, and KE_iEDV_ parameters.

**Conclusions:**

Our study suggests a potential early interaction between FT-strain and 4D-flow parameters post-STEMI leading to the development of adverse remodeling. Within the limitations of our sample size, DF and IS were independent predictors of LV remodeling after adjustment for LVEF, SV, FT-strain, and KE parameters. These findings suggest that these parameters may contribute to further risk stratification at 3M for the development of adverse remodeling at 12M post-STEMI, above conventional LV function parameters. Larger studies are needed to confirm these results.

## Graphical abstract

**Figure:**
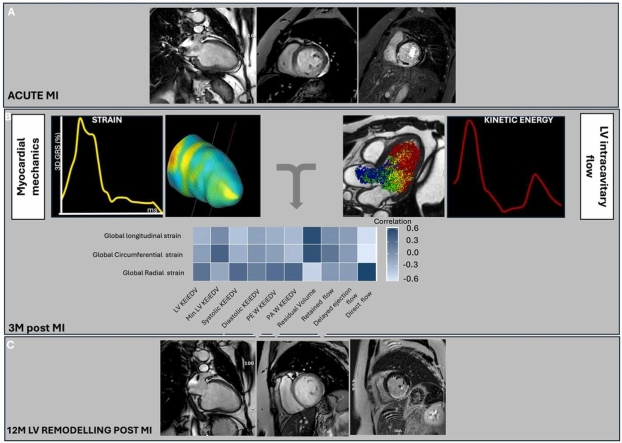
(A) Two-cavity and short-axis view of an illustrative patient showing transmural LGE in the mid and apical inferior segment in the acute setting. (B) Illustration of the myocardial mechanistics and LV intracavitary flow at 3 months post STEMI. Direct flow at 3 months post STEMI was significantly correlated with global feature tracking parameters. (C) Two-cavity and short-axis view of an illustrative patient showing LV remodelling at 12 months post STEMI. *LGE* late gadolinium enhancement, *LV* left ventricular, *STEMI* ST elevation myocardial infarction;

## 1 Introduction

The myocytes are organized in layers with different orientations, wrapping around the ventricle [1]. The highly sophisticated interplay between these myofiber layers results in highly efficient cardiac pump function, and thus in adequate left ventricular (LV) flow kinetics. Following acute myocardial infarction (MI), a sudden drop of the LV contractile function leads to increased LV pressure and volume overload and thus, to wall stress. Failure to normalize the wall stress leads to longitudinal changes of the myocardium over time, called adverse myocardial remodeling. In clinical practice, LV ejection fraction (LVEF) and/or stroke volume (SV) are the only indices of contractility used to diagnose and risk stratify patients with heart failure; while widely available and easy to assess, LVEF is a global measure and does not provide any insight into the structure of the heart and its relation to the myocardial function. The exact mechanisms underlying the cardiac maladaptive changes post MI are not fully understood; however, it is very likely that alteration of the myocardial contractility post MI leads to alteration of LV blood flow kinetics, thus contributing to the progressive LV chamber dilation and failure of the LV systolic function [2]. In recent years, research has begun to focus on the potential emerging role of fluid-structure interaction as main cause of LV remodeling. This approach is now possible thanks to state-of-the-art imaging techniques such as cardiovascular magnetic resonance (CMR), which allows to accurately characterize not only the tissue composition and myocardial deformation or strain [3] post MI, but also of the intracavitary flow [3], [4], [5]. Studies have demonstrated the potential clinical utility of CMR assessment of myocardial deformation and/or intracavitary flow quantification.

Specifically, research has demonstrated the incremental prognostic value of global longitudinal strain (GLS) and global circumferential strain (GCS) as assessed by feature tracking (FT) CMR early after acute MI for mortality over and above LVEF and infarct size (IS) [6], [7], [8], [9]. Four-dimensional flow (4D-flow) CMR imaging allows semi-automatic three-dimensional (3D) quantification of intra-cavity LV flow kinetic energy (KE) and proportions of blood flowing in and out the LV cavity at different time points in the cardiac cycle. [10] The alteration of the LV blood flow KE parameters following MI has been demonstrated in a recent study [11]. These alterations are marked in patients with reduced LVEF over time [5]. Moreover, a significant inverse association of blood flow kinetic energetics with adverse LV-remodeling has been found up to 12 months after acute MI [12], [13]. Even if the incremental prognostic value of FT strain and 4D flow parameters post MI has been well demonstrated in previous studies, the relationship between myocardial strain and LV flow kinetic in the development of adverse myocardial remodeling has not yet been fully investigated.

Adverse LV remodeling post MI is considered one of the main determinants of long-term outcomes as it can lead to heart failure and death [14]. The changes in LV structure and shape can be very dynamic, especially in the earlier stage post-acute event. Recent data show that the majority (64%) of patients post MI experience adverse LV remodeling during the first 3 months [15] with the rest of patients developing remodeling up to 12 months. Additionally, Ottervanger et al. demonstrated that among ST-elevation MI (STEMI) patients with LVEF ≤40% on the third day after MI, one-quarter demonstrated an LVEF improvement to >40% 6 months after MI [16]. Despite published data showing how LVEF is not a good discriminator and does not reliably stratify patients [17], [18] current guidelines [19], recommend risk stratification for major events at 3 months post MI based on the changes in LVEF. There is a clinical need for novel biomarkers for risk stratification.

Therefore, we sought to investigate the existing interaction between tissue mechanics and the intracavitary flow at 3 months post-acute MI, as well as their impact on the development of adverse myocardial remodeling at 12 months post MI.

## 2 Materials and methods

The study protocol was approved by the institutional research ethics committee and complied with the Declaration of Helsinki. All participants gave written informed consent for their participation (National Institute of Health Research study no. 33963 and Research Ethics no. REC 17/YH/0062). The participation to this study did not induce any delay in the standard of care clinical management.

### 2.1 Patient population

Patients who had a “first event” STEMI were prospectively recruited from a single tertiary center. Inclusion criteria were (1) MI as defined by current international guidelines [19], (2) revascularization via percutaneous coronary intervention (PCI) within 12 h after onset of symptoms, and (3) no contraindications to CMR. Exclusion criteria were (1) previous revascularization procedure (coronary artery bypass grafts or PCI), (2) known cardiomyopathy, (3) severe valvular heart disease, (4) atrial fibrillation, and (5) haemodynamic instability lasting longer than 24 h following PCI and contraindications to CMR. All patients underwent standard of care clinical management as recommended by contemporary guidelines [20].

### 2.2 Cardiovascular magnetic resonance imaging protocol

**Fig. 1:**
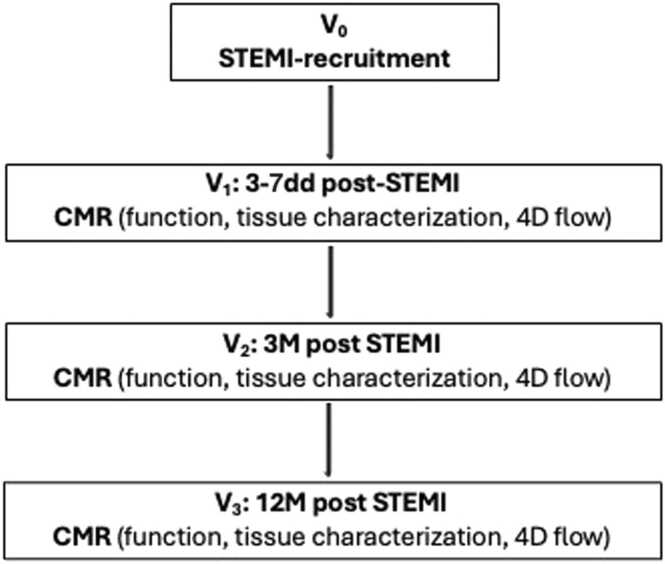
Overview of the study timeline and assessments. V_0_ represents STEMI onset. CMR with feature tracking (FT) and 4D flow kinetic assessment was performed at V_1_ (3–5 days post-STEMI), V_2_ (3 months post-STEMI), and V_3_ (12 months post-STEMI). Remodeling groups were defined based on changes between the acute (V_1_) and 12 months (V_3_) CMR. *4D* four-dimensional, *STEMI* ST-elevation myocardial infarction, *CMR* cardiovascular magnetic resonance

Participants underwent a CMR examination within 3–7 days after acute STEMI (visit 1: V1), at 3 months (visit 2: V2) and 12months (visit 3: V3) post STEMI (Fig. 1). CMR examinations were performed with a 3.0T scanner (Achieva TX, Philips Healthcare, Best, The Netherlands) equipped with a 32-channel cardiac phased array receiver coil, MultiTransmit technology and high-performance gradients with Gmax = 80 mT/m and slew rate = 100 mT/m/ms. The cardiac magnetic resonance imaging protocol included full LV coverage with functional cine and late gadolinium enhancement (LGE) imaging, and 4D flow acquisitions as previously described [21].

#### 2.2.1 Acquisition of CMR functional parameters

Cine images were acquired using a breath-hold balanced steady-state free precession (bSSFP) pulse sequence and included: two, three, four-chamber views as well as LV volume contiguous short-axis stack. Specific parameters for bSSFP were as follows: echo time (TE)/repetition time (TR)/flip angle 1.3 ms/2.6 ms/40°; spatial resolution 1.6 × 2.0 × 10 mm; typical temporal resolution 25 ms; slice thickness 8 mm. Thirty phases per cardiac cycle were reconstructed.

#### 2.2.2 4D flow acquisition

A field of view (FOV) was planned in the trans-axial plane while ensuring complete LV coverage. 4D flow data were acquired using a 3D echo-planar imaging (EPI)-based, fast field echo pulse sequence with retrospective electrocardiogram-gating. No respiration motion correction was performed and breath-hold was not mandatory. The acquisition voxel size and the reconstructed voxel size were 3 × 3 × 3 mm^3^ and 2.23 × 2.23 × 3 mm^3^. Specific parameters for 4D flow acquisition were as follows: FOV = 400 × 300 mm^2^, TR = 8.1 ms, TE = 3.5 ms, flip angle = 10°, number of signal averages = 1, velocity encoding = 150 cm/s, EPI factor (k-space profiles/excitations) = 5. These acquisition parameters allowed for the reconstruction of 30 phases across the cardiac cycle. Quality controls of the acquired images were performed as previously published [3].

#### 2.2.3 LGE imaging

LGE imaging was performed at 15 min after gadolinium-based contrast injection, using phase-sensitive inversion recovery (PSIR) spoiled gradient echo sequence. PSIR sequence parameters were as follows: sensitivity encoding (SENSE) factor 1.7, typical TE/TR of 3.0/6.1 ms, flip angle of 25°, slice thickness of 10 mm, and with Look-Locker scout determined T1-inversion time.

### 2.3 CMR image analysis

Cvi42 software (Circle Cardiovascular Imaging Inc., Calgary, Alberta, Canada) was used to assess LV volumes, LVEF, and 3D FT strain from functional images as well as the IS from LGE images.

Adverse LV remodeling (LV_remod_) was defined by a ≥20% increase in LV end-diastolic volume indexed for body surface area (BSA) (LVEDVi) [6] at 12 months post STEMI from acute setting, in opposition to the non-remodeling group (LV_non-remod_).

The threshold used for identifying infarcted tissue was set to 5 standard deviations (SD) above remote myocardial tissue signal intensity on LGE images.

**Fig. 2:**
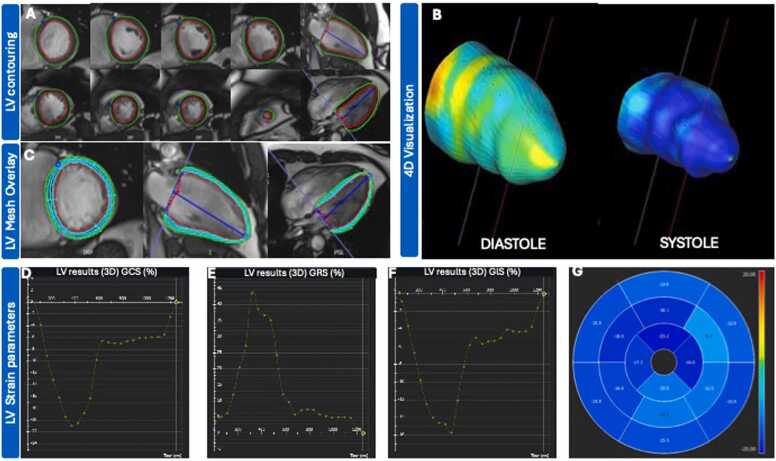
Assessment of FT strain parameters. (A) Illustration of the manual contouring of the endocardial and epicardial borders in the end-diastolic frame for all short- and long-axis slices. Right ventricular insertion points were also manually defined within the LV. (B) 3D deformation model obtained from short- and long-axis images. (C) Illustration of LV mesh overlay in short- and long-axis images for quality control of tracking and segmentation. Illustration of the global results for: GCS (D), GRS (E), GLS (F) as well as the segmental GLS results (G) *FT* feature tracking, *LV* left ventricular, *GCS* global circumferential strain, *GRS* global radial strain, *GLS* global longitudinal strain

*Feature tracking analysis (*Fig. 2*)*.

FT measurements were derived from CMR performed at 3 months post STEMI.

3D FT strain using LV cine short-axis stack, cine 2 and 4 chambers views, as previously validated [22], [23], was performed to obtain GRS, GCS, and GLS (Fig. 2). For this purpose, the endocardial and epicardial borders were manually delineated in the end-diastolic frame (defined as the cardiac phase with the largest LV volume) for all short- and long-axis slices, ensuring identical end-diastolic phases across all slices within a subject. Furthermore, right ventricular insertion points within the LV were defined in the short-axis slices. The LV outflow tract as well as the apical segments were excluded from the analysis. 2D CMR-FT tracks reference points on the mid myocardial wall over the cardiac cycle in short-axis or long-axis cine images to obtain a deformable model of the myocardium. Information on both short- and long-axis images was combined to obtain a 3D deformation model of the myocardium, enabling quantification of myocardial strain globally or segmentally in radial, circumferential, and longitudinal directions. The accuracy of FT was manually verified by assessing the tracking of the endocardial and epicardial borders. Quality control of tracking and segmentation was conducted using software tools such as mesh, boundaries, or myocardial points. In cases of tracking issues, delineation was retraced and adjusted. Segments with persistent tracking issues were excluded from analysis.

**Fig. 3:**
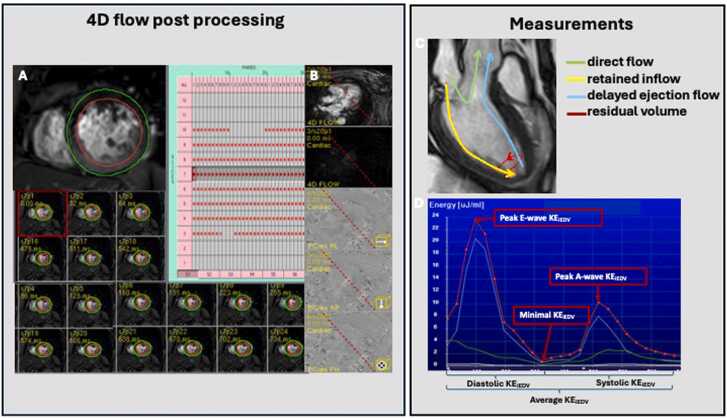
(A, B) Illustration of the 4D flow post processing. (A) Illustration of the manual contouring of the endocardial and epicardial borders. The segmentation was performed for all short-axis cine images. (B) Registration process between short-axis cine images and 4D flow data to correct for any patient movement between acquisitions. (C, D) Illustration of the 4D flow components: (C) direct flow (green line): blood flowing into the LV during diastole and exiting the LV during systole in the examined cardiac cycle. Retained volume (yellow line): blood flowing into the LV during diastole and not exiting the LV during systole in the examined cardiac cycle. Delayed ejection (flow (blue line): blood starting and remaining within the LV during diastole, and exiting during systole. Residual volume (red line): blood remaining within the LV for a minimum of two cardiac cycles. (D) LV blood flow KE curves during the cardiac cycle. Red curve represents LV endocardium. Blue curve represents basal LV myocardial segments. Green curve represents mid LV myocardial segments. White curve represents apical LV myocardial curves. *Minimal KE_iEDV_* average KE of the LV flow at any time point during the whole cardiac cycle, *Average KE_iEDV_* average KE of the LV flow at any time point during the whole cardiac cycle, *Systolic KE_iEDV_* average KE of the LV flow during the systole, *Diastolic KE_iEDV_* average KE of the LV flow during the diastole, *Peak E-wave KE_iEDV_* peak KE of the LV flow during early diastolic filling, *Peak A-wave KE_iEDV_* peak KE of the LV flow during late diastolic filling, *4D* four-dimensional, *LV* left ventricular

*4D flow analysis (*Fig. 3*)*.

4D flow measurements were derived from the CMR acquisition at 3 months post STEMI.

4D flow data were assessed using the research software tool MASS (Leiden University Medical Center, Leiden, The Netherlands). The KE parameters were calculated in a time-resolved manner, and the analysis was based on time-resolved 3D segmentation of the LV blood pool. Specifically, the LV blood pool was segmented from the short-axis cine images, which provided dynamic, volumetric data over the cardiac cycle. To ensure accurate motion tracking, a registration process was applied between the short-axis cine images and the 4D flow data to correct for any patient movement that may occur between acquisitions. Volumetric measures included: residual volume, retained inflow, delayed ejection,direct flow. Regarding the calculation of KE, the time-resolved 3D LV segmentation allowed us to capture the entire LV volume at each time point during the cardiac cycle, and KE was computed throughout this volumetric 3D model. This method allows for a comprehensive assessment of KE across the entire LV volume rather than relying on slice-based or planar KE calculations. For the reported KE parameters (minimal, average, systolic, diastolic, peak E- and A-wave), the integration of KE values was performed across the entire LV volume at each time point, and these were averaged over the cardiac cycle or specific phases (e.g., systole, diastole, E-wave, A-wave) to derive the final metrics. This approach avoids the need for combining planar KE values from individual short-axis slices, as the volumetric data inherently captures the necessary dynamic behavior of the LV blood pool throughout the cardiac cycle [3]. A description of the 4D flow parameters is given in Fig. 3.

### 2.4 Statistical analysis

Normality of the data distribution was assessed using the Shapiro–Wilk test. Continuous variables were reported as mean ± SD or median ± interquartile range depending on the variable distribution. Comparison between variables was performed using chi-square, parametric (Student’s t test) or non-parametric (Mann–Whitney) statistical test as appropriate. Correlations between KE and FT strain parameters at 3 months were assessed using Spearman correlation analysis. Univariate and multivariate logistic analyses were performed to identify predictors of adverse remodeling at 12 months. To mitigate the effect of dimensionality due to limited number of cases with remodeled LV, to identify the most significant predictors of LV remodeling, both forward and backward selection methods based on Akaike Information Criterion were employed. To ensure the stability and validity of our model, the potential of the remaining predictors to act as confounders was assessed by examining whether their inclusion would significantly alter the regression coefficients of the most significant predictors. Cross-validation to assess the generalizability of our findings was performed. All tests were assumed to be statistically significant when p < 0.05. Statistical analyses were performed in SPSS (version 29.0, Statistical Package for the Social Sciences, International Business Machines, Inc., Armonk, New York ).

## 3 Results

### 3.1 Patient characteristics

As shown in Table 1, 49 patients (M:F = 26:13, age 58 ± 10 years) with full CMR dataset acquired at 3 months post STEMI were included in this study; out of these, 23 participants (18 male and 5 female) developed adverse LV remodeling at 12 months. There were no significant differences between LV_non-remod_ and LV_remod_ participants. In both groups, the culprit coronary vessels were the left atrial descending coronary artery (47%) and the right coronary artery (45%) in most patients.

**Table 1:** Patient characteristics.

	All (n = 49)	LV_non-remod_ (n = 26)	LV_remod_ (n = 23)	p-value
*Baseline demographics*				
Sex, M/F	49	18: 8 (53%)	18:5 (47%)	0.532
Age, y	58±10	57±9	59±12	0.591
BMI, kg/m^2^	27±5	28±16	27±4	0.665
Time interval from STEMI to V1, d	5±2	6±3	5±2	0.721
Time interval from STEMI to V2, d	95±15	97±18	95±15	0.377
Time interval from STEMI to V3, d	372±23	371±18	380±45	0.229
*Cardiovascular risk factors*				
Hypertension	13	7 (14%)	6 (12%)	1.000
Positive family history	18	10 (20%)	8 (16%)	1.000
Diabetes mellitus	10	6 (4%)	4 (4%)	0.731
Current smoker	15	9 (18%)	6 (12%)	0.552
*Cardiovascular history*				
History of PVD	2	2 (4%)	0 (0%)	0.491
History of CVD	2	2 (4%)	0 (0%)	0.491
*Culprit territory*				
Left main stem	0	0	0	1.000
Left anterior descending	23	9	14	0.089
Left circumflex	4	2	2	1.000
Right coronary	22	15	7	0.085
*Medication post STEMI*				
Aspirin	49	26	23	1.000
Adenosine diphosphate receptor antagonist (Ticagrelor)	49	26	23	1.000
ACE inhibitor or angiotensin-II receptor blocker	49	26	23	1.000
Beta-blocker	48	25	23	1.000

Values are given in mean ± SD or median ± IQR. Percentages in brackets represent the percentage of the corresponding parameter for all the participants. p-value assessed the difference between the LV_no-remod_ and LV_remod_ group.

*ACE* angiotensin converting enzyme, *BMI* body mass index, *CVD* cardiovascular disease, *LV_non-remod_* no adverse remodeling at 12 months, *LV_remod_* adverse remodeling at 12 months, *PVD* peripheral vascular disease, *STEMI* ST elevation myocardial infarction, *V1* timepoint of the first CMR scan, *V2* timepoint of the second CMR scan, *V3* timepoint of the third CMR scan, *LV* left ventricular, *CMR* cardiac magnetic resonance, *SD* standard deviations, *IQR* interquartile range

### 3.2 CMR measurements

Participants in the LV_remod_ group had a significantly lower EF and higher IS in comparison to LV_non-remod_ group (EF: 41 ± 10 vs 53 ± 7%, p < 0.001; IS: 27 ± 10 vs 10.6 ± 11%, p < 0.001) (Table 2). Between the groups, there was no significant difference in SV which remain normal [24], even after indexing for BSA. All FT strain parameters were significantly reduced in the LV_remod_ group compared to the LV_non-remod_ group (GRS: 16.3 ± 7 vs 23.5 ± 7.6, p < 0.001; GCS: −12.2 ± 2.7 vs −15.1 ± 1.6, p < 0.001; GLS: −9.4 ± 3 vs −12.4 ± 2, p < 0.001). Within the 4D flow biomarkers, systolic KEi_EDV_ was significantly reduced in the LV_remod_ group (7.3 ± 2.1 µJ/mL in LV_remod_ vs 9.3 ± 3.4 µJ/mL in LV_non-remod_, p = 0.03). There was no significant difference between both groups for the remaining KE_iEDV_ parameters. Additionally, the residual volume was significantly higher in the LV_remod_ group (37.7 ± 17.7% vs 22.5 ± 10.1%, p < 0.001); the direct flow was significantly lower in the LV_remod_ group (20.7 ± 7.3% vs 35.3 ± 8.2%, p < 0.001) while the retained inflow was at the limit of significance between both groups (22 ± 6.4% vs 18.6 ± 6%, p = 0.05). There was no significant difference between both groups for the delayed ejection flow.

**Table 2:** Global and group-specific CMR parameters of the participants.

CMR parameters	All (n = 49)	No adverse remodeling at 12 months (n = 26)	Adverse remodeling at 12 months (n = 23)	p-value
*Global CMR parameters*				
EDV (mL)	158±16	144±28	185±67	0.002
EDVi (mL)	82±27	74±12	91±23	<0.001
ESV (mL)	80±41	67±16	119±63	<0.001
SV (mL)	74±16	75±18	73±13	0.992
SVi (mL/m^2^)	38±7	38±8	38±6	0.764
LVEF (%)	47±11	53±7	41±10	<0.001
Infarct size (IS) (%)	18±12	10.6±11	27±10	<0.001
*FT strain parameters*				
GRS	20.2±7.4	23.5±7.6	16.3±7	<0.001
GCS	−13.8±2.6	−15.1±1.6	−12.2±2.7	<0.001
GLS	−11±2.9	−12.4±2	−9.4±3	<0.001
*4D flow-derived KEi_EDV_ parameters (µJ/mL)*				
LV KEi_EDV_	7.3±2.7	7.4±5.1	6.7±2.1	0.213
Minimal LV KEi_EDV_	0.9±0.5	1±0.5	0.8±0.7	0.882
Systolic KEi_EDV_	7.5±3.9	9.3±3.4	7.3±2.1	0.018
Diastolic KEi_EDV_	6.9±3.6	6.8±7.4	6.8±3	0.723
Peak E-wave KEi_EDV_	16.3±12.3	17±13	15.8±9.4	0.249
Peak A-wave KEi_EDV_	12±10.7	14.3±11.2	10.7±9.6	0.184
*4D flow-derived intracavitary volumes (in %)*				
Residual volume	27.6±19.2	22.5±10.1	37.7±17.7	<0.001
Retained volume	20.3±6.3	18.6±6	22±6.4	0.05
Delayed ejection flow	21.2±7.2	21.4±7.8	21±6.6	0.802
Direct flow	28.5±10.7	35.3±8.2	20.7±7.3	<0.001

Values are given in mean ± SD or median ± IQR. p-value assessed the difference between the no-adverse-LV-remodeling group and the adverse-LV-remodeling group.

*GCS* global circumferential strain, *GLS* global longitudinal strain, *GRS* global radial strain, *CMR* cardiovascular magnetic resonance, *EDV* end-diastolic volume, *ESV* end-systolic volume, *SV* stroke volume, *SVi* stroke volume index. *LVEF* left ventricular ejection fraction, *FT* feature tracking, *4D* four-dimensional, *LV* left ventricular, *Minimal KE_iEDV_* average KE of the LV flow at any time point during the whole cardiac cycle, *Average KE_iEDV_* average KE of the LV flow at any time point during the whole cardiac cycle, *Systolic KE_iEDV_* average KE of the LV flow during the systole, *Diastolic KE_iEDV_* average KE of the LV flow during the diastole, *Peak E-wave KE_iEDV_* peak KE of the LV flow during early diastolic filling, *Peak A-wave KE_iEDV_* peak KE of the LV flow during late diastolic filling, *SD* standard deviations, *IQR* interquartile range

### 3.3 Relationship between KE and FT strain parameters

**Fig. 4:**
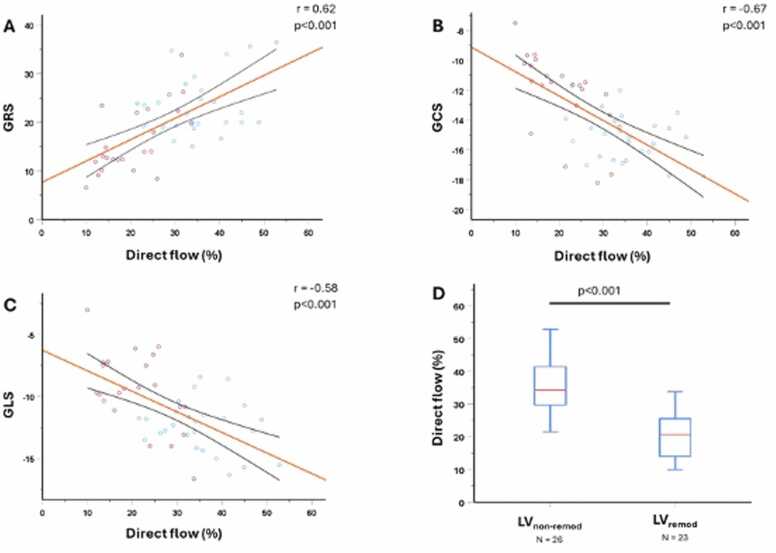
Correlations between direct flow and myocardial deformation at 3 months. (A) GRS vs direct flow (%); (B) GCS vs direct flow (%); (C) GLS vs direct flow (%). (D) Subgroup analysis of 4D flow-derived direct flow between LV_remod_ and LV_non-remod_ patients. Patients prone to develop adverse cardiac remodeling at 12 months post STEMI (blue circle) had significantly lower percentage of direct flow at 3 months post STEMI in comparison to those without adverse cardiac remodeling at 12 months post STEMI (red circle). *GCS* global circumferential strain, *GLS* global longitudinal strain, *GRS* global radial strain, *LV_remod_* adverse left ventricular remodeling at 12 months post STEMI, *LV*_*non-remod*_ no adverse left ventricular remodeling at 12 months post STEMI, *STEMI* ST-elevation myocardial infarction, *4D* four-dimensional

**Fig. 5:**
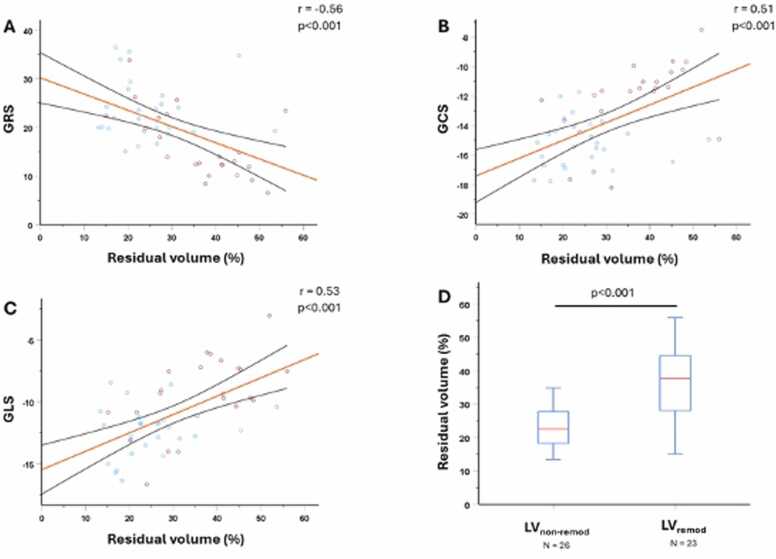
Correlation between residual volume and myocardial deformation at 3 months. (A) GRS vs residual flow, (B) GCS vs residual flow, (C) GLS vs residual flow. (D) Subgroup analysis of 4D flow-derived residual flow between LV_remod_ and LV_non-remod_ patients. Patients prone to develop adverse cardiac remodeling at 12 months post STEMI (blue circle) had significantly higher percentage of residual volume at 3 months post STEMI in comparison to those without adverse cardiac remodeling at 12 months post STEMI (blue circle). *GCS* global circumferential strain, *GLS* global longitudinal strain, *GRS* global radial strain, *LV_remod_* adverse left ventricular remodeling at 12 months post STEMI, *LV_non-remod_* no adverse left ventricular remodeling at 12 months post STEMI, *STEMI* ST-elevation myocardial infarction, *4D* four-dimensional

To understand the existing relation between myocardial mechanics and intraventricular flow, we assessed the correlations between myocardial deformation and intracavitary flow markers. As from Table 3, the markers of myocardial strain correlated significantly with Peak-A-wave KE_iEDV_ (GRS: r = 0.38, p = 0.008; GCS: r = −0.30, p = 0.038; GLS: r = −0.29, p = 0.04), direct flow (GRS: r = 0.62, p < 0.001; GCS: r = −0.67, p < 0.001; GLS: r = −0.58, p < 0.001) (Fig. 4) and residual volume (GRS: r = −0.56, p < 0.001; GCS: r = 0.51, p < 0.001; GLS: r = 0.53, p < 0.001) (Fig. 5). Systolic KE_iEDV_ significantly correlated with GRS (r = 0.31, p = 0.033) and GLS (r = −0.35, p = 0.012) and not with GCS (r = −0.2, p = 0.172).

**Table 3:** Correlation between FT strain parameters and 4D flow-derived KE as well as 4D flow-derived volume parameters, using the Spearman correlation.

	LV KEi_EDV_	Min. LV KEi_EDV_	Systolic KEi_EDV_	Diastolic KEi_EDV_	PEW KEi_EDV_	PAW KEi_EDV_	Residual volume	Retained volume	Delayed EF	Direct flow
*GRS*										
Correlation	0.07	−0.23	0.31	0.01	0.22	0.38	−0.56	−0.004	−0.08	0.62
p-value	0.656	0.111	0.033*	0.939	0.124	0.008*	<0.001*	0.980	0.610	<0.001*
*GCS*										
Correlation	0.11	0.39	−0.20	0.16	−0.08	−0.30	0.51	0.24	−0.04	−0.67
p-value	0.452	0.005*	0.172	0.278	0.603	0.038*	<0.001*	0.09	0.809	<0.001*
*GLS*										
Correlation	−0.11	0.08	−0.35	−0.003	−0.17	−0.29	0.53	−0.002	−0.001	−0.58
p-value	0.445	0.579	0.012*	0.985	0.246	0.04*	<0.001*	0.99	0.99	<0.001*

*EF* ejection flow, *GCS* global circumferential strain, *GLS* global longitudinal strain, *GRS* global radial strain, *Min* minimum, *PAW* peak A-wave, *PEW* peak E-wave, *Minimal KE_iEDV_* average KE of the LV flow at any time point during the whole cardiac cycle, *Systolic KE_iEDV_* average KE of the LV flow during the systole, *Diastolic KE_iEDV_* average KE of the LV flow during the diastole, *LV* left ventricular, *FT* left ventricular, *4D* four-dimensional

Data are correlation coefficients and p-values.

* Significant correlation (at the 0.05 level)

In the univariate analysis, all FT strain parameters as well as the direct flow, the residual volume, and systolic KE_iEDV_ were predictive of adverse remodeling at 12 months. The retained inflow parameter was not predictive of adverse remodeling at 12 months. After adjustment for FT strain parameters, KE and 4D flow parameters as well as LVEF, SV, and IS, only direct flow and IS were independent predictors of adverse remodeling at 12 months (Table 4).

**Table 4:** Univariable and multivariable logistic regression analysis for prediction of adverse LV-remodeling at 12 months.

	Univariate analysis			Multivariate analysis		
	OR	95% CI	p-value	OR	95% CI	p-value
GRS	0.84	[0.74–0.94]	0.002	ns		
GCS	1.78	[1.28–2.48]	<0.001	ns		
GLS	1.59	[1.19–2.12]	0.002	ns		
Direct flow	0.78	[0.68–0.89]	<0.001	0.804	[0.67–0.99]	0.039
Residual volume	1.12	[1.04–1.20]	0.002	Ns		
Systolic KEi_EDV_	0.76	[0.60–0.97]	0.028	Ns		
LVEF	0.85	[0.78–0.94]	<0.001	Ns		
IS	1.19	[1.08–1.30]	<0.001	1.15	[1.01–1.30]	0.032

*GCS* global circumferential strain, *GLS* global longitudinal strain, *GRS* global radial strain, *IS* infarct size, *LVEF* left ventricular ejection fraction, *Ns* non significative, *Systolic KE_iEDV_* average KE of the LV flow during the systole, *OR* odds ratio, *CI* confidence interval, *LV* left ventricular

Data are: odd ratio, confidence interval, p-values

## 4 Discussion

The dynamic interplay over time between myocardial deformation and intracavitary flow following acute STEMI requires further investigations, since it remains incompletely understood. Our study first assesses the interaction between global FT strain and 4D flow parameters measured at 3 months post-acute STEMI as well as their prediction of adverse LV remodeling at 12 months post STEMI. The main findings of our study are

1. at 3 months post STEMI, there is a significant correlation between global FT strain parameters and 4D flow parameters
2. at 3 months post STEMI, out of all FT strain and 4D flow parameters, direct flow is the only independent predictor of adverse remodeling at 12 months after adjustment for FT strain and KE parameters as well as marker of the infarct severity such as SV, LVEF, and IS.
3. at 3 months, direct flow is more accurate in discriminating LV_remod_ from LV_non-remod_ in comparison to conventional LV function parameters such as LVEF and SV.

There is an intrinsic link between intracavitary blood flow and myocardial deformation. Indeed, intracavitary blood motion results from a well-orchestrated myocardial systolic and diastolic function. Thus, intracavitary blood flow at different time of the cardiac cycle reflects the integrity of the cardiac motion. This close relationship allows for the estimation of flow forces inside the cardiac chambers through a thorough understanding of tissue motion [25]. Moreover, a previous investigation using speckle tracking and intraventricular flow analysis assessed by echocardiography demonstrated a significant correlation between GLS and both energy dissipation kinetic and energy fluctuation indexes [26].

There is accumulating evidence on the utility of FT strain and KE parameters in the risk stratification of patients post STEMI. The prognostic value of deformation/strain biomarkers measured acutely in MI patients has been previously reported for major adverse cardiovascular event [8] and for LV remodeling [7]. In keeping with previously published data, in our present study, all FT strain parameters at 3 months post STEMI were found to be significantly lower in the group prone to develop adverse cardiac remodeling at 12 months.

We previously demonstrated a reduction in LV blood flow KE_iEDV_ parameters post MI [11] and in patients with adverse remodeling [5] at 12 months. Demirkiran et al. demonstrated the independent predictive value of diastolic KE parameters for the development of adverse cardiac remodeling at 3 months post STEMI [12].

To the best of our knowledge, this study is the first one looking at the interaction between mechanical deformation and intracavitary flow in patients post MI using CMR. While our findings related to FT strain and flow separately are mostly in keeping with previously published literature [5], [11], [12], [13], systolic KE_iEDV_ and all the diastolic KE_iEDV_ parameters as well as all FT strain parameters at 3 months post MI were not able to independently predict adverse cardiac remodeling at 12 months post STEMI, in contrary to previous investigations [7], [12]. We might speculate whether the small size of our study population compared to previous investigations or the shorter follow-up time in previous study (3 months in [12] vs 9 months in our study) might be the reason for this discrepancy. As shown in Table 3, our study shows that Min. LV KEi_EDV_ correlates with GCS, indicating that LV stiffness and compliance at end-diastole are primarily influenced by circumferential mechanics. Systolic KEi_EDV_ correlates with GRS and GLS, reflecting their role in the ejection phase. In contrast, PAW KEi_EDV_ correlates with all strain parameters, as atrial contraction is influenced by both active myocardial mechanics and diastolic function. However, PEW KEi_EDV_, associated with myocardial relaxation, does not correlate with strain measures, highlighting the passive nature of relaxation during early diastole [27]. These findings may provide new insights into the distinct contributions of myocardial strain during different phases of the cardiac cycle. Another strength of our study relies on the fact that it focusses on the reassessment of the LV function at 3 months post MI, as recommended by current guidelines [28] to further risk-stratify patients prone to develop adverse cardiac remodeling at 12 months. Above standard CMR LV function metrics, novel 4D flow parameters such as direct flow could play a role in this patient re-stratification. The significant correlation between FT strain parameters and direct flow, as shown in Fig. 3, confirmed the interplay between myocardial mechanistic and intracavitary flow motion. Therefore, in patients prone to adverse remodeling at 12 months, the reduction in FT strain at 3 months correlated significantly with a change in intracavitary systolic KE and direct flow which were not depicted by SV (Table 2). Indeed, in our study, there was no significant difference in the SV at 3 months post MI between patients prone to develop adverse LV remodeling at 12 months and those not. A previous study investigated the utility of direct flow in comparison to SV in patients with dilated cardiomyopathy (DCM) in comparison to healthy participants [29]. There was no significant difference between SV in the two groups. However, direct flow was significantly lower in the DCM group in comparison to healthy population. Despite being well compensated, as shown by the preserved SV, the small proportion of blood that transits the LV in a single cardiac cycle (direct flow) will significantly diminish in DCM patients in comparison to healthy participants. In our study, we also observed that the compensated state at 3 months post MI of patients prone to develop LV remodeling at 12 months, as demonstrated by the preserved SV, is not predictive of the LV remodeling at 12 months.

Similar to Das et al. [13], we observed a significantly reduced direct flow and increased residual volume in the LV_remod_ group. This observation confirms the utility of these parameters not only in the acute setting, but even at 3 months post STEMI. Interestingly, in addition to the investigations of Das et al. [13], our present study demonstrated the independent prognostic value of direct flow assessed at 3 months post STEMI in the prediction of adverse remodeling at 12 months, after adjustment for FT strain and 4D flow parameters as well as LVEF and IS. This finding is in line with previous evidence showing the link between the type of LV blood flow and the geometrical adaptation of the myocardial architecture [30], [31]. Indeed, the ability of LV endothelial cells to sense changes in LV loading conditions through alterations of the shear stress, resulting in adaptive responses known as mechano-transduction, was demonstrated [30]. This relationship has been observed during embryonic heart morphogenesis [31]. Following the mechano-transduction–induced adaptive response, myocardial stretching leads to various intracellular signaling pathways, resulting in decreased initial LV load [32]. Over time, this short-term highly effective mechanism leads to maladaptive myocardial remodeling. The independent prognostic value of direct flow over FT strain parameters, as observed in the present study, may provide valuable insights for constructing predictive models that may anticipate the appearance of adverse LV remodeling following acute STEMI.

## 5 Limitations

Our study has limitations. It is a single-center study, using a single vendor scanner and single field strength (3T). Our relatively small sample size (49 patients) was also a limitation. Thus, to enhance generalizability, future research should involve larger cohorts from multiple centers. Moreover, taking into account our exclusion criteria, our results are not applicable to patients with significant valvopathy, cardiomyopathies, or congenital heart disease.

## 6 Conclusion

Following MI, the early interaction between changes in myocardial deformation as assessed by FT and intracavitary flow could potentially lead to the development of long-term adverse remodeling. In this study, among all the FT strain and 4D flow parameters at 3 months post STEMI, direct flow was the only independent predictor of adverse LV remodeling at 12 months, outperforming conventional parameters for LV function assessment such as LVEF and SV. As such, 4D flow assessment in addition to standard CMR imaging might further contribute to the risk stratification of patients post STEMI, supporting the earlier intensification of heart failure therapy for patients at higher risk of adverse remodeling. These findings warrant further validation in larger studies.

## Funding

C.H.K. is supported by the development fund of the Lausanne University Teaching Hospital. S.P. is supported by 10.13039/501100000274British Heart Foundation CH/16/2/32089. The study received support from the British Heart Foundation FS/13/71/30378.

## Author contributions

**Erica Dall'Armellina:** Writing – review & editing, Validation, Supervision, Funding acquisition, Formal analysis, Conceptualization. **Rob J. Van der Geest:** Writing – review & editing, Validation, Supervision, Methodology, Investigation, Formal analysis. **Christel H. Kamani:** Writing – original draft, Software, Methodology, Investigation, Formal analysis, Data curation, Conceptualization. **Mehak Asad:** Visualization, Writing – review & editing. **Ioannis Botis:** Writing – review & editing, Formal analysis. **Noor Sharrack:** Writing – review & editing, Formal analysis. **May Lwin:** Writing – review & editing, Formal analysis. **Arka Das:** Writing – review & editing, Formal analysis. **Hadar Schapira:** Writing – review & editing, Formal analysis. **Sven Plein:** Writing – review & editing, Supervision, Conceptualization. **Peter P. Swoboda:** Writing – review & editing, Formal analysis.

## Declaration of competing interests

The authors declare that they have no known competing financial interests or personal relationships that could have appeared to influence the work reported in this paper.

## Data Availability

The datasets generated during and/or analyzed during the current study are available from the corresponding author on reasonable request.
